# Those Who Hear Music: Three Cases on Musical Hallucinations

**DOI:** 10.1155/2018/9361382

**Published:** 2018-06-27

**Authors:** Yasira Doluweera, Chathurie Suraweera

**Affiliations:** ^1^National Hospital of Sri Lanka, Sri Lanka; ^2^Faculty of Medicine, University of Colombo, Sri Lanka

## Abstract

Musical hallucination carries no diagnostic significance on its own. However, it is an interesting phenomenon which can occur in various organic and psychiatric disorders. We report three patients who presented with musical hallucinations due to different aetiologies, namely, due to hearing impairment, intracerebral haemorrhage, and schizophrenia. The case series also highlights the fact that different aetiologies should be managed differently for the patients to be benefited.

## 1. Introduction

Musical hallucinations are rare but fascinating phenomena in psychiatry which have not been explored adequately. A musical hallucination is a type of auditory hallucination where music is perceived without an external source. It is observed in primary psychotic illness, in sensory deprivation states like hearing impairment and organic psychosis.

## 2. Case One

A 78-year-old widow presented with hearing of songs for a duration of six months. She identified them as vocals with the background music of a popular singer whom she adored as a young girl. Initially it was not disturbing and she enjoyed it. However, the music became very disturbing as it got louder with time. She reported gradual hearing impairment for 2 years. In neurological examination bilateral hearing impairment was detected which was confirmed to be sensorineural auditory impairment on audiography. Her mental state examination was normal with no other psychotic phenomena or cognitive impairment.

The consultant otolaryngologist recommended her hearing aids after which the hallucinations disappeared completely without any psychotropics. One year following the intervention, she remains asymptomatic.

## 3. Case Two

A 29-year-old female was referred by the neurosurgical team as she complained of hearing music for one-week duration. She was admitted with the complaints of severe headache of sudden onset and was found to have intraventricular and intracranial haemorrhages. Following the surgical evacuation of the haemorrhages she made a full recovery. Hearing of music started one week following the surgery. She was hearing familiar songs in increased volume with distorted sounds. She had no hallucinations of any other modalities and nor there were any delusions. Her consciousness was clear and she was oriented in time, place, and person. There were impairments in her short-term memory and long-term memory along with frontal lobar impairment. She had no neurological deficits in the physical examination. A Noncontrast CT (NCCT) brain following the onset of musical hallucinations revealed no abnormalities. She was started on quetiapine 25 mg and was gradually titrated up to 150mg for which she responded. One year following the initiation of treatments she remains symptom free.

## 4. Case Three

An 86-year-old lady complained of being persecuted by her neighbours for 3-month duration. She reported that 2 males are living inside her body controlling her activities. She heard persistent voices chanting “pirith” (a form of religious sermon) for a duration of 2 years. The sounds were slow and rhythmic in nature. She found great relief with the voices and believed that she has achieved a higher spiritual status. In addition, she had persecutory delusions, delusions of control, and somatic hallucinations. Her cognitive functions were normal. She was diagnosed with very late onset schizophrenia and was commenced on risperidone 2mg in the night which was gradually increase to 5mg in the night. Even though her symptoms gradually improved, she regretted it as she no longer heard “pirith” chanting. She had no hearing impairment. One year following the initiation of treatments she has no symptoms.

## 5. Discussion

Musical hallucinations are a form of auditory hallucinations characterised by hearing of songs, tunes, and melodies in the absence of auditory stimuli [1]. This phenomenon was first described by Ballinger in 1846 and had been subsequently identified as a rare psychopathology as there are only less than 500 reported cases worldwide [1].

As opposed to other forms of auditory hallucinations, musical hallucinations are perceived as a pleasant experience by most patients [2]. This was observed in two of the patients we reported. The patient who heard religious phrases in the form of “pirith” found comfort in her music and regretted getting treatment when her symptoms improved with treatments.

In most reported cases, musical hallucinations were found to have some personal significance to the patients [3]. In a previously reported case in Sri Lanka by Weerasundara et al., 2013, a patient heard music that was played in the wedding day 50 years ago [4]. This was observed in one of our patients we reported as she heard songs by a popular singer she used to listen as a young girl. With the music, pleasant memories resurfaced and she enjoyed experience of hallucinations as a result.

This personal significance and enjoyable nature of the hallucinations have cast doubt about the true nature of the musical hallucinations. Hence, Keshavan et al., in 1992, argued that musical hallucinations are due to memories that patient find difficult to unlearn [5]. However, contrary to what Keshavan reported, all three patients displayed the characteristics of true hallucinations as opposed to other perceptual abnormalities like pseudo hallucinations and auditory echo similar to most other reported cases.

Musical hallucinations are known to have heterogeneous aetiologies. Hearing impairment, psychosis, organic conditions including epilepsy, brain tumours, head injury, encephalitis, multiple sclerosis, and substance intoxication are among the commonest causes. Golden et al., in 2015, identified causes of musical hallucinations in a sample of 393 patients identified by the Mayo Medical Record Linkage System [6]. In this sample 39% had a psychiatric diagnosis, 25 % had a neurological basis, and in 15% of cases a clear cause was not identified.

The number of cases with auditory Charles Bonnet Syndrome with hearing impairment has been reported. Lesions anywhere in the auditory tract leading to hearing impairment can lead to musical hallucinations [7]. Release phenomenon is a theory that tries to explain musical hallucinations in those with deafness. This theory states that deafness through sensory deprivation to the auditory cortex leads to spontaneous activity to occur in the auditory pathway causing musical hallucinations [7].

Structural brain lesions can cause musical hallucinations [6]. It has been proposed that musical hallucinations occur more commonly in nondominant lobe pathologies than the dominant lobe as the former is responsible for the musical perception than the latter [8]. However, this lateralisation hypothesis has been questioned in recent studies as temporal lobe involvement was noted in many cases indicating its role in sound perception [9]. One of the patients in this series had an intraventricular and intracranial bleeding. In her NCCT brain there was bilateral medial temporal lobe involvement.

Musical hallucinations are a rare form of psychopathology in primary psychotic disorders. In a survey by Golden et al. in 2015 the majority of the patients with psychiatric causality for musical hallucinations had depression followed by bipolar affective disorder. Schizophrenia was found in 2% of patients [6]. Contrary to the musical hallucinations caused by other aetiologies, musical hallucinations caused by psychosis tend to be unpleasant. Our patient with schizophrenia had persistent musical hallucinations in the form of chanting of “pirith”. As opposed to reported cases she enjoyed her chanting and regretted the disappearing following treatments.

There is a paucity of evidence on treatment of musical hallucinations. Treating the aetiology has been known to remit musical hallucinations in some cases. Auditory Charles Bonnet Syndrome is known to remit following the improvement of hearing [10]. Case studies report successful management of musical hallucinations using antidepressants, antipsychotics, and cognitive enhancers depending on the aetiology [11]. The prognosis depends on the aetiology. Our patient with auditory Charles Bonnet Syndrome did not improve when the hearing was improved. However, other two patients with intracranial haemorrhage and schizophrenia improved with antipsychotics.

There is a deficiency of literature on this fascinating phenomenon. Further research is needed to look for the aetiology, neurobiology, and treatment of this rare but unique psychopathology.  Table 1 shows the comparison of the characteristics of the three patients.

## Figures and Tables

**Table 1:** Comparison of the characteristics of the three patients.

	Case 1	Case 2	Case 3
Demographic Features	78-year-old widow	29-year-old female garment factory worker	86-year-old devoted Buddhist female

Songs of Hallucinations	One song she first heard and liked as a young girl 50 years ago	3-4 current songs with no special preference	chanting “pirith” (a form of religious sermon)

Accompanying music	Accompanying music present	Accompanying music present	No accompanying music

Amplitude	Initially low amplitude but became louder with time causing distress	High amplitude to a level of distress	Slow, rhythmic chanting of the same amplitude.

Memories associated	Pleasant memories of the past	No associated memories	No associated past memories

Associated Emotions	Enjoyed the hallucination	Was distressed with the hallucinations	Pleasant emotions were evoked as a result of voices

Diagnosis	Auditory Charles Bonnet Syndrome	Organic Psychotic disorder	Very late onset schizophrenia

Treatments	Hearing aids	Quetiapine 25mg	risperidone 2mg

Response to treatments	Hallucinations completely resolved with treatment.	Symptoms improved with treatments.	Significant improvement of symptoms and the patient regretted the disappearance of hallucinations
