# Corrigendum to “Those Who Hear Music: Three Cases on Musical Hallucinations”

**DOI:** 10.1155/2021/7603280

**Published:** 2021-01-30

**Authors:** Yasira Doluweera, Chathurie Suraweera

**Affiliations:** ^1^National Hospital of Sri Lanka, Sri Lanka; ^2^Faculty of Medicine, University of Colombo, Sri Lanka

In the article titled “Those Who Hear Music: Three Cases on Musical Hallucinations” [1], there is a mistake in paragraph 9 in Discussion. The following section should be corrected from “Our patient with auditory Charles Bonnet Syndrome did not improve when the hearing was improved. However, other two patients with intracranial haemorrhage and schizophrenia improved with antipsychotics.” to “Our patient with auditory Charles Bonnet Syndrome did improve when the hearing was improved. However, other two patients with intracranial haemorrhage and schizophrenia improved with antipsychotics.”
